# Antiviral Activity of Nitazoxanide and Miltefosine Against FeHV-1 In Vitro

**DOI:** 10.1155/2024/8849561

**Published:** 2024-10-19

**Authors:** Consiglia Longobardi, Gianmarco Ferrara, Sara Damiano, Salvatore Florio, Giuseppe Iovane, Ugo Pagnini, Serena Montagnaro, Roberto Ciarcia

**Affiliations:** University of Naples Federico II, Via Federico Delpino, 1, Naples 80137, Italy

**Keywords:** antiviral, FeHV-1, herpesvirus, miltefosine, nitazoxanide, PI3K/Akt

## Abstract

Feline herpesvirus type 1 (FeHV-1) is a primary pathogen in cats responsible for respiratory and ocular signs. There are presently no antiviral drugs that are officially licensed for veterinary use in several countries. Consequently, veterinarians must depend on off-label antivirals designed for human use. Recent advances in virus–host cell interaction have resulted in new insights into FeHV-1 replication, establishing the importance of the PI3K/Akt axis. The aim of this study was to employ this new information to assess the efficacy of two compounds whose activities involve this pathway. The antiviral properties of miltefosine and nitazoxanide were examined using seven different concentrations, evaluating cell viability and viral titers after 24 h of infection. Furthermore, selected concentrations were supplied at different time points to investigate the influence of the timing of the addition. The best results were obtained when the drugs were added both before and after viral adsorption (in particular for nitazoxanide). Each compound was further investigated by real-time PCR, western blot, and immunofluorescence. Nitazoxanide was the most effective treatment, reducing the expression of viral glycoproteins as measured by western blot and immunofluorescence, as well as reducing the release of virions in the supernatant (measured by real-time PCR). Moreover, treatment with nitazoxanide and miltefosine was associated with a decrease in Akt phosphorylation. This work emphasized the significance of comprehending the pathways necessary for viral replication and their use in the assessment of novel and effective antivirals.

## 1. Introduction

Feline herpesvirus type 1 (FeHV-1) is a common pathogen responsible for respiratory and ocular disease in felids (domestic cats and wild or captive felids, including lions, cheetahs, and pumas) that was first isolated in 1958 and is now distributed worldwide. This double-stranded DNA virus belongs to the family Herpesviridae, subfamily Alphaherpesvirinae, and genus *Varicellovirus* and constitutes a single serotype [1–3]. Transmission occurs between healthy and infected cats by the oral or nasal route, whose conjunctivae are highly colonized by the virus. Clinical signs, mainly self-limiting within a couple of weeks, include fever, sneezing, ocular and nasal discharges, depression, hypersalivation, and cough [3]. However, in more susceptible animals (unvaccinated kittens or debilitated animals), the infection can be more serious, leading to severe conjunctivitis, stomatitis, and respiratory syndromes, as well as other concomitant infections (feline calicivirus, *Bordetella* spp., and *Chlamydophila* spp.) [3]. Being an enveloped and not particularly resistant virus, FeHV-1 ensures its survival in the host through the latency mechanism. During the latency, which could be lifelong, viral DNA is distributed in the trigeminal ganglia, waiting for viral reactivation caused by different kinds of stress (other infections, immunodepression, administration of corticosteroids, etc.) [4–6]. Viral reactivation can cause the recrudescence of respiratory disease and viral shedding [7]. Latency and reactivation processes are responsible for both the wide spread of this virus and the incomplete protection offered by currently available commercial vaccines [8].

In recent years, research has identified new potential antiviral drugs (theoretically targeting each stage of the viral replication process, from viral adsorption to viral release from the host cell) that have been effective (in vitro and/or in *vivo*) in mitigating the consequences caused by FeHV-1 infection [8–10]. Examples of drugs that have proven to be effective include famciclovir and its active metabolite penciclovir (although their actual effectiveness is still debated due to the absence of large-scale clinical trials), whereas acyclovir, used for HSV-1 infection in humans, is highly toxic in cats when systemically administered [11–14]. Trifluridine, idoxuridine, and vidarabine have also been proposed in the past, but their effectiveness has not been definitively confirmed. Moreover, specific antivirals licensed for veterinary use, such as cidofovir, are available only in some countries [9]. Nowadays, given the absence of formulations for specific veterinary use, off-label drugs (such as famciclovir) are used for the antiviral treatment of FeHV-1 [8]. However, these antiviral drugs have several kinds of adverse effects, including reduced appetite, vomiting, and diarrhea, all of which can be lethal in a weakened cat [9].

Several factors must be considered when assessing the efficacy of an antiviral in vitro and, especially, in vivo. While most antiviral drugs have some efficacy against FeHV-1, their safety in cats cannot be predicted based on their performance in other hosts. Similarly, the efficacy against FeHV-1 cannot be defined based on the effectiveness against other viruses, including the human herpes simplex virus type 1 (HSV-1) [12, 15]. For example, the safety of antivirals in humans does not necessarily imply their safety in animals, due to variations in metabolic processes. To date, the most successful antiviral compounds target viral DNA synthesis, and their safety mostly relies on the ability to selectively inhibit only viral DNA [16, 17].

In recent years, the scientific literature has widely discussed the antiviral properties of two drugs: nitazoxanide and miltefosine. Nitazoxanide, or 2-(acetyloxy)-N-(5-nitro-2-thiazolyl) benzamide, has been known since 1970, when it was developed as an oral antiparasitic agent. It is characterized by a niclosamide base replacing a benzene ring and is effective against several protozoa (*Giardia* spp. and *Cryptosporidium* spp.) and helminths (intestinal cestodes) [18, 19]. Nowadays, nitazoxanide has emerged as the drug of choice for the treatment of the *Cryptosporidium parvum* infection in humans. In recent years, several in vitro studies have shown that nitazoxanide exhibits a broad spectrum of antiviral action (including respiratory syncytial virus, parainfluenza virus, coronavirus, rotavirus, norovirus, hepatitis B virus, hepatitis C virus, dengue virus, yellow fever virus, Japanese encephalitis virus, and human immunodeficiency virus (HIV)), encouraging efforts to employ nitazoxanide and other thiazolides as a new class of antiviral drugs [20–24]. The efficacy of this drug has also recently been demonstrated against feline calicivirus (often identified in FeHV-1 positive animals) [25]. Miltefosine, initially developed as an antineoplastic agent, has emerged as a secondary therapeutic option for the treatment of leishmaniosis in animals [26, 27]. Although it is currently used as an antiprotozoal agent, in recent years, it has also been proposed as an antiviral treatment against HSV-1 and HIV [28, 29]. Information on the interactions between FeHV-1 and host cells has been recently updated [30–32]. Recent work has demonstrated how the PI3K/Akt/mTOR axis undergoes modifications during FeHV-1 infection and particularly during viral entry [31, 32]. FeHV-1 phosphorylates Akt to enter host cells. Inhibitors that target Akt phosphorylation have been found to effectively decrease FeHV-1 entry, while silencing Akt is not effective in this process (probably due to the Us3 kinase encoded by the virus). The purpose of this work is therefore to evaluate the antiviral activity against FeHV-1 of two drugs able to act on this axis: nitazoxanide and miltefosine.

## 2. Materials and Methods

### 2.1. Cells, Virus, and Drugs

The FeHV-1 strain Ba/91 (provided by Prof. C. Buonavoglia, School of Veterinary Medicine of Bari) and its permissive cells (Crandell-Rees Feline Kidney Cells, CRFK) cultured in 25 cm^2^ flasks were used. Briefly, CRFK (CRFK CCL-94, ATCC) were maintained in Dulbecco's modified Eagle's medium (DMEM; Corning) supplemented with 10% fetal bovine serum (FBS). When 90% confluent, cells were infected with a Multiplicity of Infection (MOI) of 0.5 (this viral dosage was selected since it can cause an evident cytopathic effect (CPE)). After 1 hour of viral adsorption (at 37°C) in serum-free medium, infected and control cells were washed with 1x phosphate-buffered saline (PBS), incubated in fresh DMEM for 24 h, and monitored for CPE. Two different compounds with antiviral activity were used in this study. Nitazoxanide (Sigma) was used at doses of 0.5, 1.5, 5, 20, 80, 100, and 200 *μ*M, as reported in a previous study [25]. Miltefosine (Sigma) was used in concentrations of 0.01, 0.05, 0.1, 0.25, 1.5, 2.5, and 5 *μ*M [29].

### 2.2. Evaluation of Antiviral Effects

After incubation with the virus (MOI 0.5) and different concentrations of drugs, the supernatants of several experimental conditions were collected and used in viral titration assay using the Reed–Muench method [33]. Each experiment was carried out three times. The data obtained with CRFK cells were used to calculate the 50% efficacy concentration (EC_50_) (carried out using the GraphPad software).

### 2.3. Cytotoxicity Assay

The same experiments were performed in 96-well plates using the same conditions of viral dose, incubation time, and drug dose to conduct the 3-(4,5-dimethylthiazol-2-yl)-2,5-diphenyltetrazolium bromide (MTT) assay [34]. FEA cells (RRID: CVCL_UG17), a feline embryonal cell line, were also used to evaluate cytotoxicity. MTT powder (SERVA) was dissolved in Roswell Park Memorial Institute (RPMI) medium at a concentration of 0.5 mg/mL. After removing the medium from each well, 100 *μ*L of MTT solution was dispensed and incubated at 37°C for 3 hours. After further removing the contents of each well, 100 *μ*L of dimethylsulfoxide (DMSO) was added for the solubilization step. Then each plate was read using a spectrophotometer at 570 nm. Each experiment was carried out three times, and the results were represented as percentage of cell viability (using the control as reference). The data obtained with CRFK cells were used to calculate the 50% cytotoxic concentration (CC_50_) which was carried out using the GraphPad software.

### 2.4. Time Addition Assay

All drugs were added at different times during the viral infection: post (only after absorption, i.e., into the fresh medium that replaced viral absorption medium), during (only during absorption, i.e., into the serum-free medium used for the adsorption), and pre-post (3 hours before adsorption and for the 24 h following viral adsorption). These experiments were carried out in 96-well plates to carry out the MTT assay and in 6-well plates to collect the supernatants for the TCID_50_.

Doses lower than the CC_50_ that did not cause significant reductions in viability and simultaneously higher than the EC_50_ were used (20 *μ*M nitazoxanide and 0.25 *μ*M miltefosine) in the timing addition experiment.

### 2.5. Real-Time PCR

DNA was extracted from supernatants using a commercial kit (Qiagen) and quantified using a Nanodrop. Extracted DNA was used as a template for SYBR green real-time amplification of thymidine-kinase (TK) gene. Specific primers (Forward primer: 5′ TGTCCGCATTTACATAGATGG 3′; Reverse primer: 5′ GGGGTGTTCCTCACATACAA 3′) and protocols were described in a previous work [32]. Gene expression analysis was performed using a standard curve constructed with virus crude DNA (extracted from 100 *μ*L of FeHV-1 titrated 10^7^ TCID_50_ using Reed and Muench method) and read by a Bio-Rad CFX 96 Touch real-time PCR detection device.

### 2.6. Immunofluorescence

Cells grown on microscope slides were treated and infected as previously described, maintaining the viral dose, drug concentrations, and incubation time. Monolayers were washed, fixed, and incubated with an anti-FeHV-1 mouse monoclonal antibody (Novus Biologicals, targeting glycoprotein B and I) eluted 1:200 in 2% BSA. A secondary anti-mouse antibody labeled with Alexa Fluor 610 was used (Thermo Fischer Scientific) at a dilution of 1:100. The cell nuclei were stained with 4′,6-diamidino-2-phenylindole (DAPI). Each slide, appropriately mounted on a glass slide, was observed and photographed using the ZOE™ Fluorescent Cell Imager (Bio-Rad).

### 2.7. Giemsa Assay

This assay was carried out with the same conditions described previously (MOI, method of adding the drug, time of incubation, etc.), cultivating the cells in a 6-well flask. At the end of the incubation, cells were washed with 1x PBS and fixed in methanol. After another washing step with 1x PBS, cells were stained with Giemsa (Sigma) to observe CPE [34]. Images were obtained using ZOE™ Fluorescent Cell Imager (Bio-Rad).

### 2.8. Western Blot Analysis

CRFK treated pre-post with the most effective doses of the relative drugs and incubated with 0.5 MOI of FeHV-1 for 24 h were washed with 1x PBS, scraped, centrifuged, washed an additional time, and lysed with a suitable lysis buffer (RIPA containing protease and phosphatase inhibitors, Sigma) at 4°C [30]. The protein content of the cell pellet was collected and quantified by Bradford assay (Bio-Rad). A total of 25 *μ*g of each lysate (mixed with Laemmli buffer) was loaded into wells of pre-cast acrylamide gel (Bio-Rad) for electrophoresis. At the end of the run, each gel was transferred onto nitrocellulose membranes using a TransBlot Turbo (Bio-Rad). The blocking step, consisting of an hour of incubation in 5% BSA, preceded the incubation step with primary and secondary antibodies. Incubation with primary and secondary antibodies was followed by three washing steps with 1x Tween-Tris-buffered saline (TBST). The following primary and secondary antibodies have been used: FeHV-1 (Novus biological), Akt (Cell Signaling), p-Akt (Cell Signaling), *β*-Actin (Santacruz) as normalizer, and Horseradish peroxidase (HRP)–linked secondary antibodies anti-mouse and anti-rabbit (Cell Signaling). Clarity Western ECL Substrate (Bio-Rad) and a ChemiDoc Blot scanner (Bio-Rad) were used for visualization. Protein expressions were assessed by densitometric analysis using Image Lab software.

### 2.9. Statistical Analysis

Three independent replicates were carried out for each experiment. The statistical significance of the variables, expressed as the mean ± standard deviation (SD), was determined using one-way ANOVA (GraphPad Prism 6.0). A *p* value less than 0.05 was considered statistically significant (marked as ⁣^∗^), a *p* value lower than 0.01 was considered highly statistically significant (marked as ⁣^∗∗^), and a *p* value lower than 0.001 was considered extremely statistically significant (indicated as ⁣^∗∗∗^).

## 3. Results

### 3.1. Effects of Nitazoxanide and Miltefosine on Cell Viability and Virus Replication

**Figure 1:**
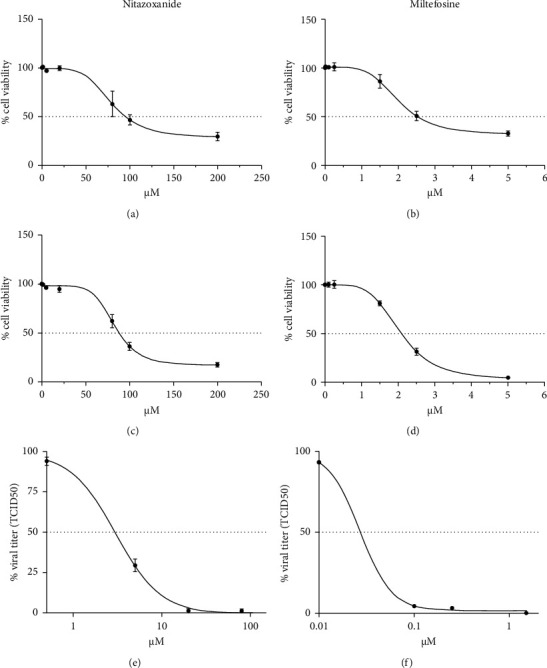
Effects on cell viability and viral titers evaluated on FeHV-1-infected CRFK cells (MOI 0.5) for 24 h and treated with different concentrations of nitazoxanide (a, e) and miltefosine (b, f). Effects on cell viability in FEA cells treated with different concentrations of nitazoxanide (c) and miltefosine (d) were also evaluated.

**Figure 2:**
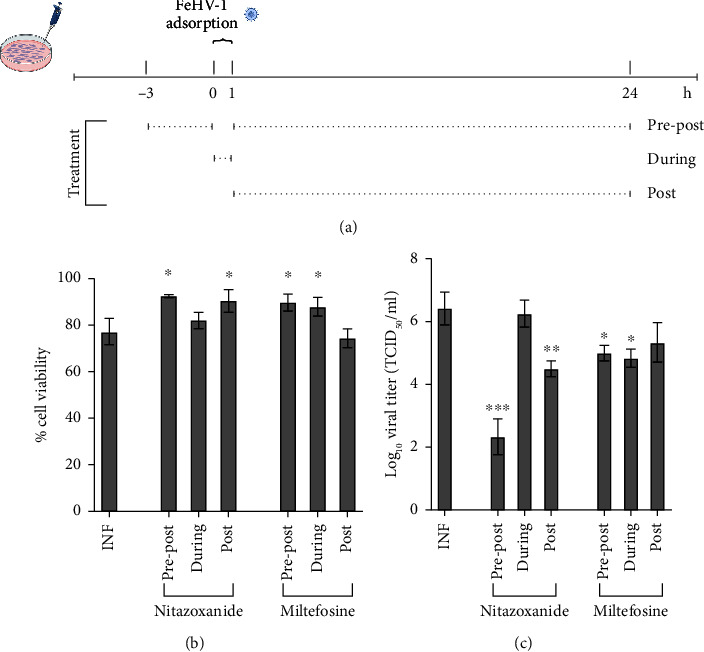
Effect of selected concentrations (a) of nitazoxanide and miltefosine on cell viability (b) and viral titers (c) during (during adsorption), before infection (pre), and both before and after (pre-post) (⁣^∗^*p* < 0.05; ⁣^∗∗^*p* < 0.01; ⁣^∗∗∗^*p* < 0.001).

To determine the CC_50_ and EC_50_ of miltefosine and nitazoxanide, the MTT assay and TCID_50_ were performed with different doses to assess the viability of treated CRFK cells and the effects on viral replication (Figure 1). The results obtained with MTT highlighted the cytotoxic effects of the two highest concentrations of both drugs (which were discarded for the evaluation of viral titers). Additionally, the cytotoxicity was further evaluated on FEA cells, showing similar results. The CC_50_s for nitazoxanide and miltefosine were 78.87 *μ*M and 2.02 *μ*M, respectively (Table 1). The evaluation of viral titers was carried out for the five non-cytotoxic doses of each drug, which established the EC_50_ of each compound (3.01 *μ*M, 0.03 *μ*M for nitazoxanide and miltefosine, respectively). The doses with the best cytotoxicity/efficacy combination were selected and used for the timing addition experiment, evaluating viability and viral titers with the addition of the drugs at various times (Figure 2(a)). Nitazoxanide reduced FeHV-1-induced cytotoxicity at a dose of 20 *μ*M when added before and after infection (pre-post), as well as when added solely after infection (post) (Figure 2(b)). Pre-post treatment with nitazoxanide proved to be the one that most significantly reduced FeHV-1 replication, reducing viral excretion by about 3 logarithms (Figure 2(c)).

In the case of miltefosine, an increase in viability was observed in infected cells at 0.25 *μ*M doses when added during adsorption (during) and when supplied before and after infection (pre-post) (Figure 2(b)). Miltefosine has shown a good ability to reduce the viral titer when added during absorption and pre-post (Figure 2(c)). The pre-post treatment resulted in a decrease in the cytotoxic activity produced by the virus for both drugs and was selected for further investigation.

### 3.2. Nitazoxanide and Miltefosine Affect FeHV-1 Replication Involving the Phosphorylation of Akt

**Figure 3:**
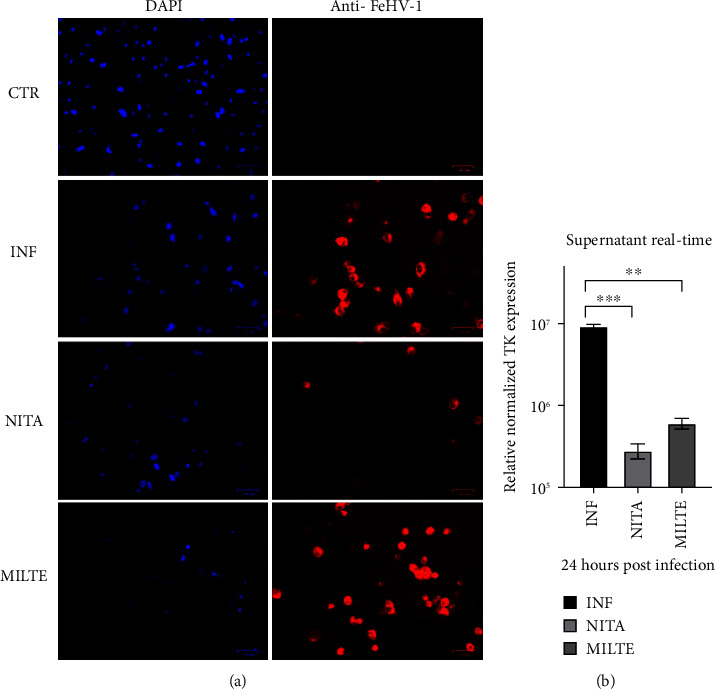
Immunofluorescence staining (a) of CRFK cells infected with FeHV-1 (INF) and treated with nitazoxanide (NITA, 20 *μ*M) and miltefosine (MILTE, 0.25 *μ*M). Control cells were used as negative controls. Red: FeHV-1; blue: DAPI. Real-time PCR (b) quantification of the TK gene on supernatants collected by CRFK infected with FeHV-1 (INF) and treated with nitazoxanide (NITA, 20 *μ*M) and miltefosine (MILTE, 0.25 *μ*M) (⁣^∗∗^*p* < 0.01; ⁣^∗∗∗^*p* < 0.001).

The final investigations (IFA, real-time PCR, Giemsa, and western blot) were conducted using the previously selected dose of each drug (20 *μ*M nitazoxanide and 0.25 *μ*M miltefosine) added pre-post. The results of the IFA supported the previously reported outcomes, indicating that the most effective suppression of viral replication was achieved by nitazoxanide (Figure 3(a)). This was evident from the lower red fluorescence (related to the expression of viral proteins by cells) that was observed in cells treated with nitazoxanide compared to infected cells (and miltefosine-treated cells).

Moreover, TK gene expression evaluated in real-time PCR was markedly reduced with the use of all the tested drugs, in particular nitazoxanide (Figure 3(b)).

**Figure 4:**
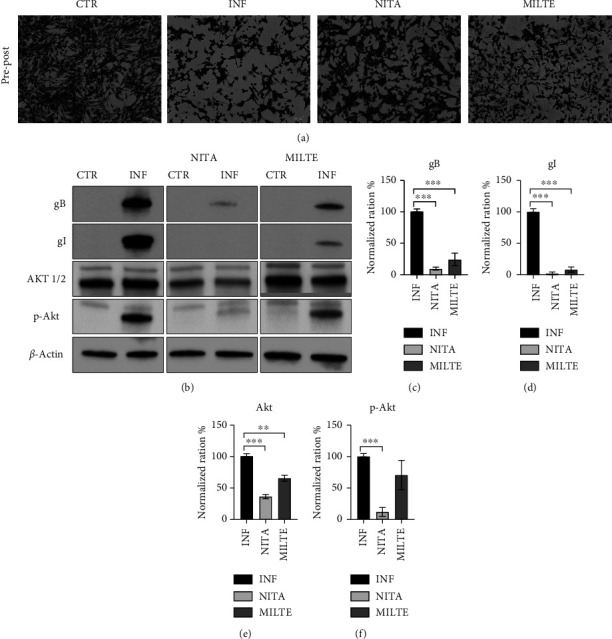
Giemsa assay (a) on CRFK cells infected with FeHV-1 (INF) and treated with nitazoxanide (NITA, 20 *μ*M) and miltefosine (MILTE, 0.25 *μ*M). Western blot analysis of viral glycoproteins and cellular Akt expressions (b). Comparison of gB (c), gI (d), Akt (e), and p-Akt (f) protein expression in CRFK cells infected with FeHV-1 (INF) and treated with nitazoxanide (NITA, 20 *μ*M) and miltefosine (MILTE, 0.25 *μ*M). Cropped images are derived from three different blots. Full blots and relative actin are available as support files (Supporting File 1).

The Giemsa assay was used to determine the amount of the CPE generated by FeHV-1 using selected dosages of each drug (Figure 4(a)). A reduced CPE was found with nitazoxanide. Although lower than nitazoxanide, miltefosine also significantly reduced the CPE of FeHV-1 on CRFK cells.

Finally, western blot analysis (Figure 4(b)) showed a statistically significant reduction of viral glycoproteins B (gB) and I (gI), more pronounced in cells treated with nitazoxanide (Figures 4(c) and 4(d)), where their expression almost disappeared. In particular, gI expression was nearly totally inhibited (Figure 4(d)). The inhibition of Akt phosphorylation also showed this trend: The use of nitazoxanide reduced the expression of constitutive Akt by over 50% and almost completely prevented its phosphorylation (Figure 4(f)). The prevention of Akt phosphorylation was less pronounced with miltefosine (Supporting File 1).

## 4. Discussion

Antivirals, classified as virostatic, cannot target latent herpesvirus infection and require frequent administration, potentially enhancing side effects. During FeHV-1 latency, the viral genome is stored in peripheral neurons in the absence of an infectious virus and is not addressable by antivirals, even if it has the capacity to reactivate infection. All of these factors significantly limit the success of antivirals in veterinary medicine [8, 9]. As a result, identifying effective and safe antivirals for the treatment of FeHV-1 is a valuable objective in order to improve pet health and enhance the fight against this virus. Several antiviral drugs designed for the treatment of human herpesviruses have also been utilized in cats. However, many of these drugs have limited effectiveness for the treatment of FeHV-1 due to poor absorption or potential risks associated with systemic administration. Other treatments, such as lysine supplementation extensively used in the past, proved to be not beneficial (according to meta-analysis data) [35]. From a clinic point of view, famciclovir and cidofovir remain the preferred options for systemic and topical administration, respectively. This preference persists despite recent findings indicating that famciclovir exhibits varying effectiveness against FeHV-1 [10, 13, 36]. In this study, we evaluated the in vitro antiviral activity of two drugs, nitazoxanide and miltefosine, currently being explored as novel antivirals despite their conventional pharmacological use. Nitazoxanide and miltefosine were initially manufactured and distributed as an antiprotozoal agent against *Cryptosporidium parvum* and *Leishmania* spp., respectively, but they were subsequently discovered to be first-in-class broad-spectrum antiviral drugs active against a wide range of viruses [19, 29]. Our results have successfully determined their optimal in vitro pharmacological doses, which are both highly effective and free of cytotoxicity. Nitazoxanide is a thiazolide used as a drug of first choice for the treatment of certain protozoal infections. Its antiviral activities and its application for the treatment of viral infections of veterinary interest have recently been discussed [37]. Recent studies have also proposed a possible mechanism of action for this compound, which could involve the depletion of intracellular calcium and an influence on protein kinase R (PKR), both mechanisms involved in the viral entry process [37]. The outcome observed in western blot analysis indicated how nitazoxanide affected FeHV-1 replication involving the PI3K/Akt axis. Furthermore, nitazoxanide markedly decreased the expression of constitutive Akt and prevented the phosphorylation of Akt, which FeHV-1 employs for its entry into the host cell, according to recent studies [31]. If validated by future research, the use of nitazoxanide might be beneficial since it targets specific ways of FeHV-1 entrance.

However, the activity of this drug may not be directed only to Akt since, according to recent evidence, specific silencing of Akt-1 proved to be poorly effective in limiting the entry of the virus due to the presence of a viral kinase called Us3 (able to act as an Akt surrogate) [38, 39]. Thanks to this kinase, the virus can phosphorylate Akt even when cellular Akt is not fully accessible (such as when inhibitors are used or during Akt silencing). For this reason, there were elements suggesting further mechanisms of action for this drug. This hypothesis is supported by the fact that viral-induced cytotoxicity and viral yield were unaffected when nitazoxanide was employed only during viral uptake.

The absence of cytotoxicity at a dose of 20 *μ*M and the simultaneous antiviral activity have already been highlighted by a recent study about the possible use of nitazoxanide for the treatment of feline calicivirus [25]. In the same study, in vivo performances were also evaluated (doses between 5 and 20 mg/kg were effective), which led to a significant reduction in viral load in the trachea and lungs and reduced viral shedding [25]. In a study conducted on the chikungunya virus, however, this compound was effective at a dose between 12.5 *μ*M and 25 *μ*M, while a lower dose (2.5 µM) resulted in effectiveness against astroviruses [20, 21].

Miltefosine's mechanism of action (employed for the inhibition of HIV and HSV-1 amplification) has also been described in the literature and includes the inhibition of Akt phosphorylation and the depletion of calcium ions [29, 40, 41]. These effects were confirmed in western blots and were correlated with a reduced expression of viral glycoproteins, gB and gI. Although the results were less intriguing when compared to those obtained with nitazoxanide, this drug has highlighted a notable activity that needs further investigation. However, this compound has already been tested on cats to determine its anti-leishmania properties, showing both safety and effectiveness [42].

Despite being the main antiviral drug used off-label for the treatment of FeHV-1, the efficacy of famciclovir has been debated in recent years [10, 11]. Some studies in the literature assert that famciclovir has no effect on the treatment of FeHV-1 in vitro, and its in vivo metabolism is complicated due to the limited activity of hepatic aldehyde oxidase. On the contrary, raltegravir, ganciclovir, and cidofovir have demonstrated notable efficacy as the most effective compounds in vitro, and evidence suggests mild improvement of clinical score during topical treatment of FeHV-1 [43, 44]. In the absence of antiviral drugs licensed for the treatment of FeHV-1, it is worth considering them as potential options for further investigations in the future [45]. Our study addressed the need to identify new drugs with potential antiviral activity against FeHV-1, as well as a better comprehension of their mechanisms of action, indications, and limitations.

## 5. Conclusion

This work described the antiviral effects of miltefosine and nitazoxanide on FeHV-1 and represented an example of targeting cellular pathways critical for viral replication. The results suggested that both compounds should be investigated further to better understand their in vivo effectiveness and cytotoxicity.

## Figures and Tables

**Table 1:** Determination of the 50% cytotoxic concentration (CC_50_), 50% efficacy concentration (EC_50_), their 95% confidence interval (95% CI), and selectivity index (SI) of miltefosine and nitazoxanide.

Compound	CC_50_ (*μ*M)	95% CI	EC_50_ (*μ*M)	95% CI	SI
Nitazoxanide	78.87	69.26–88.99	3.01	2.6–3.9	26.2
Miltefosine	2.02	1.87–2.17	0.03	0.02–0.03	67.3

*Note:* Results were obtained with the MTT assay and TCID_50_ performed at different doses.

## Data Availability

Data sharing not applicable to this article as no datasets were generated or analysed during the current study.

## Supporting Information

Supporting Data 1: full blots of Figure 4.

## References

[1] Thiry E., Addie D., Belák S. (2009). Feline Herpesvirus Infection ABCD Guidelines on Prevention and Management. *Journal of Feline Medicine & Surgery*.

[2] Amoroso M. G., Serra F., Miletti G. (2022). A Retrospective Study of Viral Molecular Prevalences in Cats in Southern Italy (Campania Region). *Viruses*.

[3] Monne Rodriguez J. M., Leeming G., Köhler K., Kipar A. (2017). Feline Herpesvirus Pneumonia: Investigations Into the Pathogenesis. *Veterinary Pathology Online*.

[4] Montagnaro S., Longo M., Pacilio M. (2009). Feline Herpesvirus-1 Down-Regulates MHC Class I Expression in an Homologous Cell System. *Journal of Cellular Biochemistry*.

[5] Egberink H., Frymus T., Hartmann K. (2022). Vaccination and Antibody Testing in Cats. *Viruses*.

[6] Townsend W. M., Jacobi S., Tai S. H., Kiupel M., Wise A. G., Maes R. K. (2013). Ocular and Neural Distribution of Feline Herpesvirus-1 During Active and Latent Experimental Infection in Cats. *BMC Veterinary Research*.

[7] Field H. J., Biswas S., Mohammad I. T. (2006). Herpesvirus Latency and Therapy-From a Veterinary Perspective. *Antiviral Research*.

[8] Gould D. (2011). Feline Herpesvirus-1. Ocular Manifestations, Diagnosis and Treatment Options. *Journal of Feline Medicine & Surgery*.

[9] Thomasy S. M., Maggs D. J. (2016). A Review of Antiviral Drugs and Other Compounds With Activity against Feline Herpesvirus Type 1. *Veterinary Ophthalmology*.

[10] Kopecny L., Maggs D. J., Leutenegger C. M., Johnson L. R. (2020). Effects of Famciclovir in Cats With Spontaneous Acute Upper Respiratory Tract Disease. *Journal of Feline Medicine & Surgery*.

[11] Thomasy S. M., Lim C. C., Reilly C. M., Kass P. H., Lappin M. R., Maggs D. J. (2011). Evaluation of Orally Administered Famciclovir in Cats Experimentally Infected With Feline Herpesvirus Type-1. *American Journal of Veterinary Research*.

[12] Groth A. D., Contreras M. T., Kado-Fong H. K., Nguyen K. Q., Thomasy S. M., Maggs D. J. (2014). In Vitro Cytotoxicity and Antiviral Efficacy against Feline Herpesvirus Type 1 of Famciclovir and Its Metabolites. *Veterinary Ophthalmology*.

[13] Cooper A. E., Thomasy S. M., Drazenovich T. L. (2019). Prophylactic and Therapeutic Effects of Twice-Daily Famciclovir Administration on Infectious Upper Respiratory Disease in Shelter-Housed Cats. *Journal of Feline Medicine & Surgery*.

[14] Thomasy S. M., Shull O., Outerbridge C. A. (2016). Oral Administration of Famciclovir for Treatment of Spontaneous Ocular, Respiratory, or Dermatologic Disease Attributed to Feline Herpesvirus Type 1: 59 Cases (2006–2013). *Journal of the American Veterinary Medical Association*.

[15] Lewin A. C., Ineck N. E., Mironovich M. A. (2023). Surveillance for Feline Herpesvirus Type 1 Mutation and Development of Resistance in Cats Treated With Antiviral Medications. *Frontiers in Veterinary Science*.

[16] Dong H., Wang Z., Zhao D., Leng X., Zhao Y. (2021). Antiviral Strategies Targeting Herpesviruses. *Journal of Virus Eradication*.

[17] Wilkes R. P., Hartmann K. (2016). Update on Antiviral Therapies. *August’s Consultations in Feline Internal Medicine*.

[18] Xu J., Xue Y., Bolinger A. A. (2023). Therapeutic Potential of Salicylamide Derivatives for Combating Viral Infections. *Medicinal Research Reviews*.

[19] Rossignol J. F. (2014). Nitazoxanide: A First-In-Class Broad-Spectrum Antiviral Agent. *Antiviral Research*.

[20] Hargest V., Sharp B., Livingston B., Cortez V., Schultz-Cherry S. (2020). Astrovirus Replication Is Inhibited by Nitazoxanide In Vitro and In Vivo. *Journal of Virology*.

[21] Wang Y. M., Lu J. W., Lin C. C. (2016). Antiviral Activities of Niclosamide and Nitazoxanide against Chikungunya Virus Entry and Transmission. *Antiviral Research*.

[22] Lokhande A. S., Devarajan P. V. (2021). A Review on Possible Mechanistic Insights of Nitazoxanide for Repurposing in COVID-19. *European Journal of Pharmacology*.

[23] Jasenosky L. D., Cadena C., Mire C. E. (2019). The FDA-Approved Oral Drug Nitazoxanide Amplifies Host Antiviral Responses and Inhibits Ebola Virus. *iScience*.

[24] Shi Z., Wei J., Deng X. (2014). Nitazoxanide Inhibits the Replication of Japanese Encephalitis Virus in Cultured Cells and in a Mouse Model. *Virology Journal*.

[25] Cui Z., Li D., Xie Y. (2020). Nitazoxanide Protects Cats From Feline Calicivirus Infection and Acts Synergistically With Mizoribine In Vitro. *Antiviral Research*.

[26] Brianti E., Celi N., Napoli E. (2019). Treatment and Long-Term Follow-Up of a Cat With Leishmaniosis. *Parasites & Vectors*.

[27] Gizzarelli M., Foglia Manzillo V., Inglese A., Montagnaro S., Oliva G. (2023). Retrospective Long-Term Evaluation of Miltefosine-Allopurinol Treatment in Canine Leishmaniosis. *Pathogens*.

[28] Musarrat F., Jambunathan N., Rider P. J. F., Chouljenko V. N., Kousoulas K. G. (2018). The Amino Terminus of Herpes Simplex Virus 1 Glycoprotein K (gK) Is Required for gB Binding to Akt, Release of Intracellular Calcium, and Fusion of the Viral Envelope With Plasma Membranes. *Journal of Virology*.

[29] Garg R., Tremblay M. J. (2012). Miltefosine Represses HIV-1 Replication in Human Dendritic cell/T-Cell Cocultures Partially by Inducing Secretion of Type-I Interferon. *Virology*.

[30] Ferrara G., Longobardi C., Sgadari M. F. (2023). Apoptosis Is Mediated by FeHV-1 Through the Intrinsic Pathway and Interacts With the Autophagic Process. *Virology Journal*.

[31] Ferrara G., Longobardi C., Damiano S., Ciarcia R., Pagnini U., Montagnaro S. (2023). Modifications of the PI3K/Akt/mTOR Axis During FeHV-1 Infection in Permissive Cells. *Frontiers in Veterinary Science*.

[32] Ferrara G., Sgadari M., Longobardi C., Iovane G., Pagnini U., Montagnaro S. (2023). Autophagy Up-Regulation Upon FeHV-1 Infection on Permissive Cells. *Frontiers in Veterinary Science*.

[33] Forte I. M., Indovina P., Montagnaro S. (2021). The Oncolytic Caprine Herpesvirus 1 (CpHV-1) Induces Apoptosis and Synergizes With Cisplatin in Mesothelioma Cell Lines: A New Potential Virotherapy Approach. *Viruses*.

[34] Montagnaro S., Damiano S., Ciarcia R. (2019). Caprine Herpesvirus 1 (CpHV-1) as a Potential Candidate for Oncolytic Virotherapy. *Cancer Biology & Therapy*.

[35] Bol S., Bunnik E. M. (2015). Lysine Supplementation Is Not Effective for the Prevention or Treatment of Feline Herpesvirus 1 Infection in Cats: A Systematic Review. *BMC Veterinary Research*.

[36] Van Der Meulen K., Garré B., Croubels S., Nauwynck H. (2006). In Vitro Comparison of Antiviral Drugs Against Feline Herpesvirus 1. *BMC Veterinary Research*.

[37] Ashiru O., Howe J. D., Butters T. D. (2014). Nitazoxanide, an Antiviral Thiazolide, Depletes ATP-Sensitive Intracellular Ca2+ Stores. *Virology*.

[38] Jacob T., Van den Broeke C., Favoreel H. W. (2011). Viral Serine/Threonine Protein Kinases. *Journal of Virology*.

[39] Connolly S. A., Jardetzky T. S., Longnecker R. (2021). The Structural Basis of Herpesvirus Entry. *Nature Reviews Microbiology*.

[40] Chugh P., Bradel-Tretheway B., Monteiro-Filho C. M. R. (2008). Akt Inhibitors as an HIV-1 Infected Macrophage-Specific Anti-Viral Therapy. *Retrovirology*.

[41] Cheshenko N., Trepanier J. B., Stefanidou M. (2013). HSV Activates Akt to Trigger Calcium Release and Promote Viral Entry: Novel Candidate Target for Treatment and Suppression. *The FASEB Journal*.

[42] Manna L., Vitale F., Reale S. (2009). Study of Efficacy of Miltefosine and Allopurinol in Dogs With Leishmaniosis. *The Veterinary Journal*.

[43] Fontenelle J. P., Powell C. C., Veir J. K., Radecki S. V., Lappin M. R. (2008). Effect of Topical Ophthalmic Application of Cidofovir on Experimentally Induced Primary Ocular Feline Herpesvirus-1 Infection in Cats. *American Journal of Veterinary Research*.

[44] Spertus C. B., Pennington M. R., Van de Walle G. R. (2019). Effects of Orally Administered Raltegravir in Cats with Experimentally Induced Ocular and Respiratory Feline Herpesvirus-1 Infection. *American Journal of Veterinary Research*.

[45] Longobardi C., Damiano S., Ferrara G. (2024). Green Tea Extract Reduces Viral Proliferation and ROS Production during Feline Herpesvirus Type-1 (FHV-1) Infection. *BMC Veterinary Research*.

