# Supplementary material for: Antiviral Activity of Nitazoxanide and Miltefosine Against FeHV-1 In Vitro
Source: Vet Med Int. 2024 Oct 19;2024:8849561. doi: 10.1155/2024/8849561 (PMC11512641; doi:10.1155/2024/8849561)
Supplement: Supporting Information — Additional supporting information can be found online in the Supporting Information section. [file 8849561.f1.pdf]

Akt-1

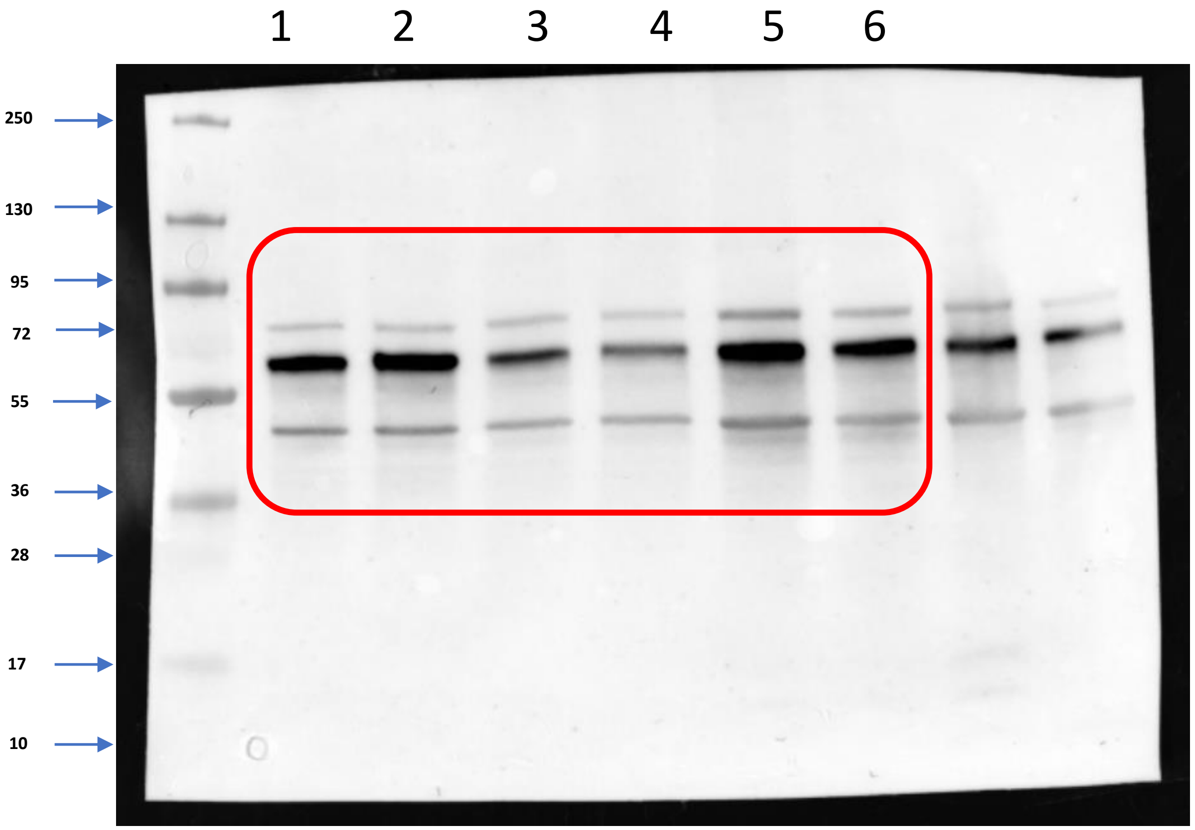

β-actin

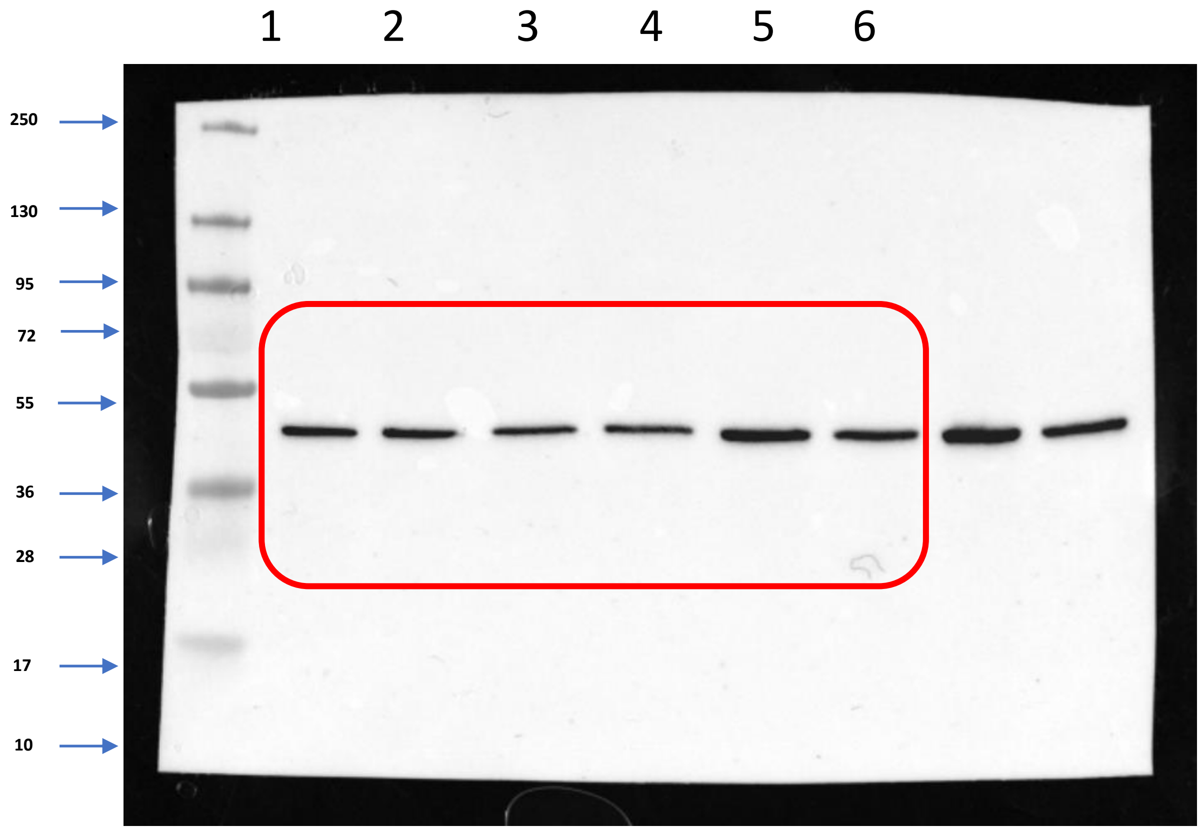

1) CTR 2)INF 3)NITA 4)NITA INF. 5)MILTE 6)MILTE INF.

Title: Whole uncropped images of the original western blots shown in figure 5

Legend: Whole uncropped images of the original western blots of Akt and β-actin.

p-Akt-1

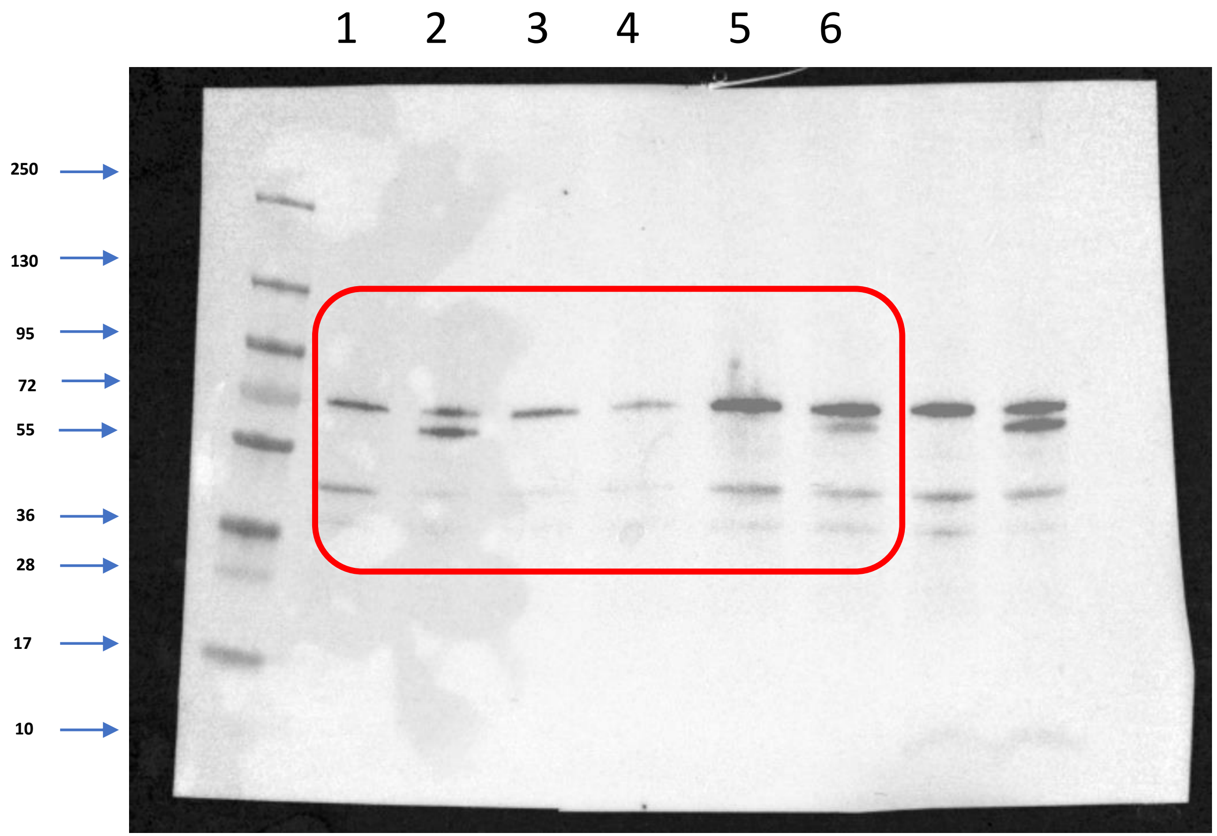

β-actin

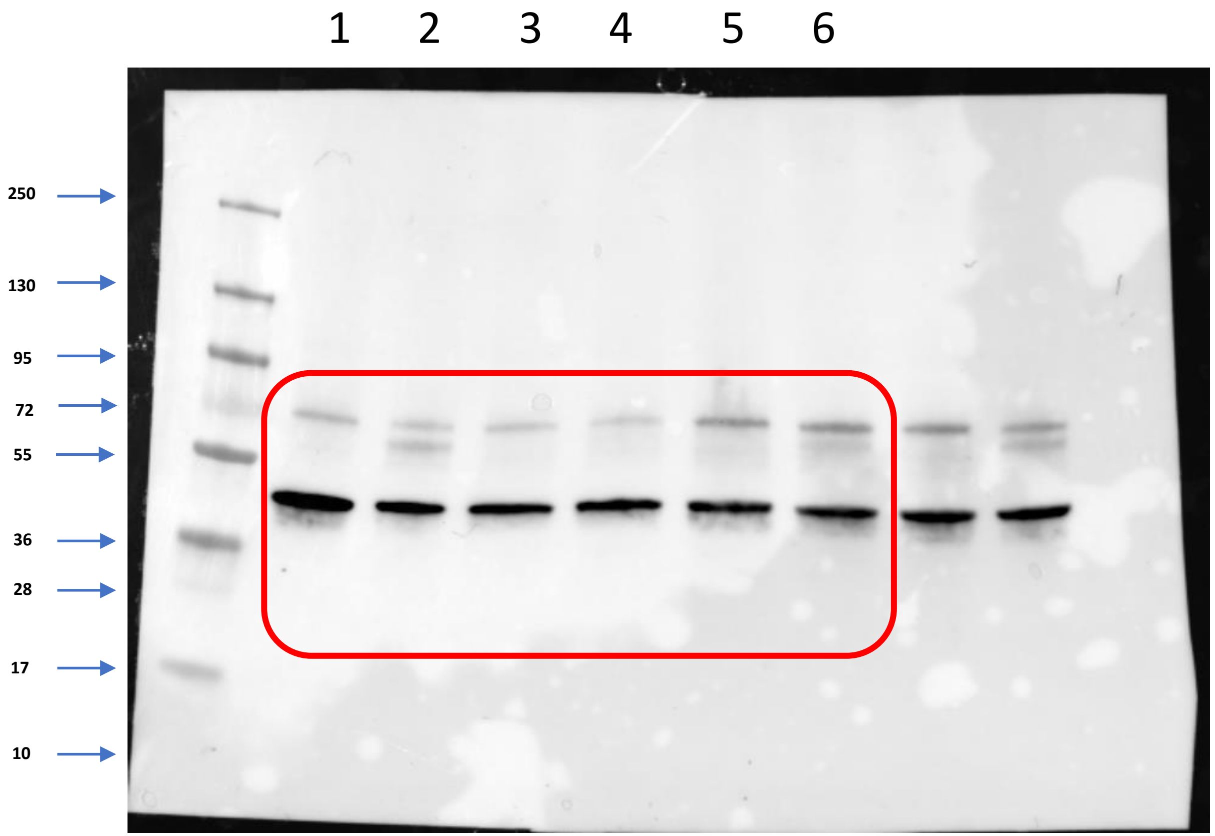

1) CTR 2)INF 3)NITA 4)NITA INF. 5)MILTE 6)MILTE INF.

Title: Whole uncropped images of the original western blots shown in figure 5

Legend: Whole uncropped images of the original western blots of p-Akt-1 and β-actin.

gB/gI

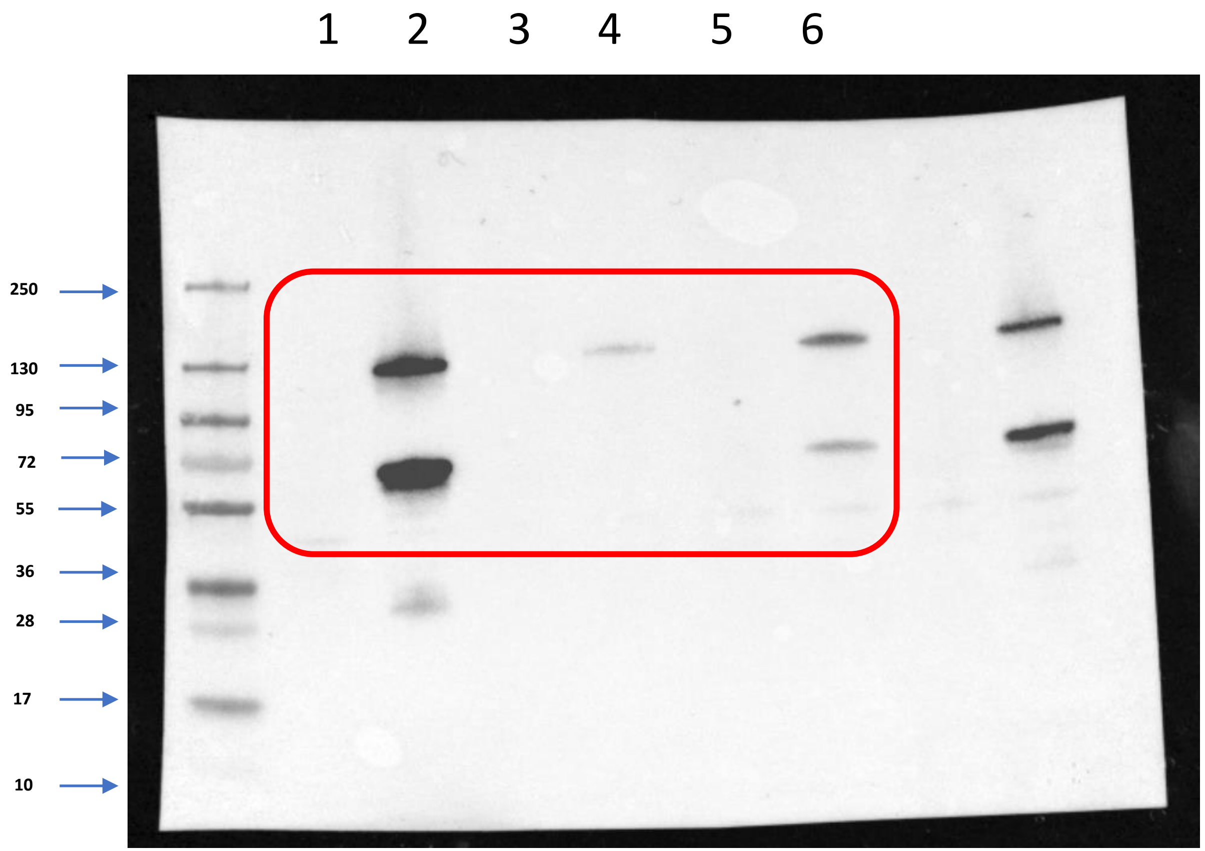

β-actin

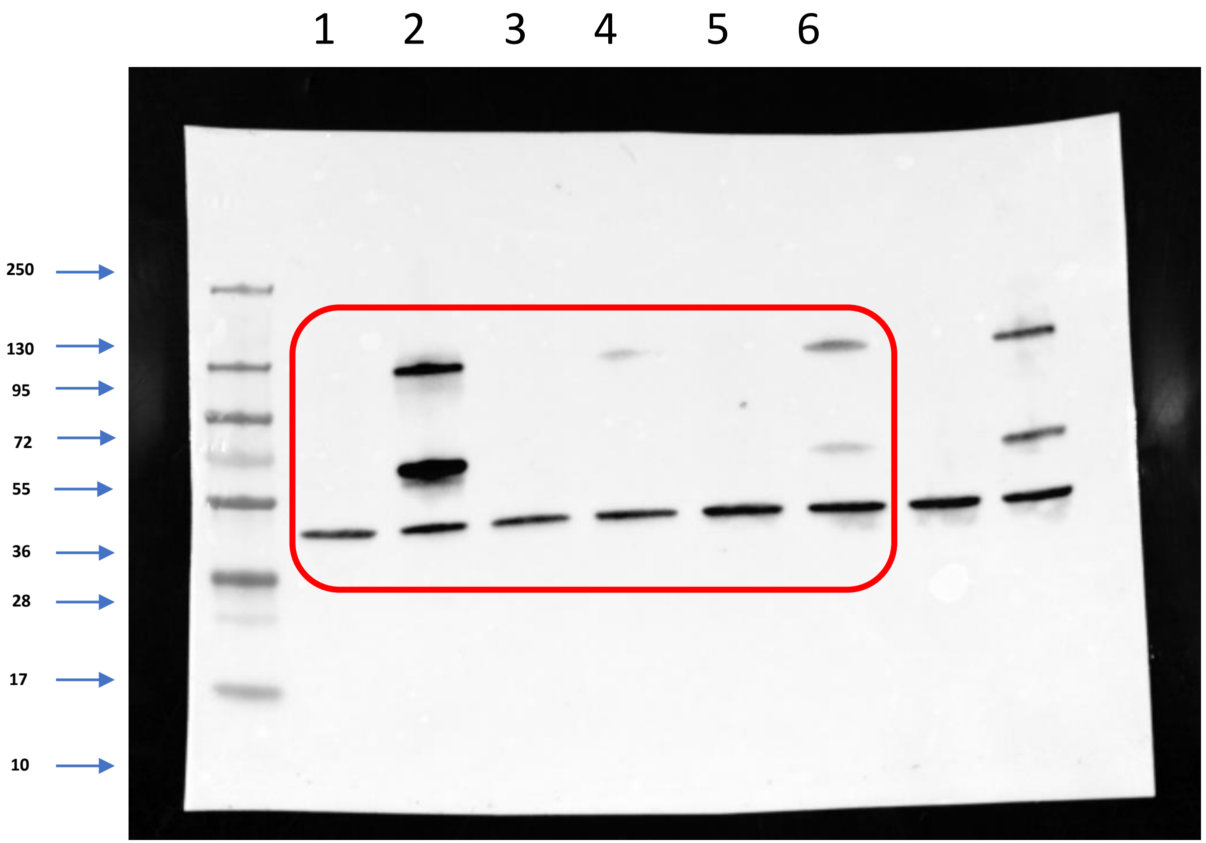

1) CTR 2)INF 3)NITA 4)NITA INF. 5)MILTE 6)MILTE INF.

Title: Whole uncropped images of the original western blots shown in figure 5

Legend: Whole uncropped images of the original western blots of gB/gI and β-actin.
